# Advanced Technology in the Management of Diabetes: Which Comes First—Continuous Glucose Monitor or Insulin Pump?

**DOI:** 10.1007/s11892-019-1177-7

**Published:** 2019-06-27

**Authors:** Christopher T. Martin, Amy B. Criego, Anders L. Carlson, Richard M. Bergenstal

**Affiliations:** 1 Allina Health, West Metro Endocrinology, Edina, MN 55435 USA; 2 International Diabetes Center, Park Nicollet Pediatric Endocrine, Minneapolis, MN 55416 USA; 3 International Diabetes Center, HealthPartners Endocrinology, Minneapolis, MN 55416 USA; 4 International Diabetes Center, HealthPartners Institutes, Minneapolis, MN 55416 USA

**Keywords:** Continuous glucose monitor, Insulin pump, Diabetes, Hypoglycemia, Time in range, Hemoglobin A1C

## Abstract

**Purpose of Review:**

In this article, we consider advanced technologies for the management of diabetes.

**Recent Findings:**

Specifically, we pose the question of which should come first: an insulin pump (CSII) or a continuous glucose monitor (CGM)? Historical perspective on both insulin delivery and glucose measurement is provided. Recently published clinical trials are reviewed. Practical issues including quality of life, patient education, and out-of-pocket cost are discussed.

**Summary:**

Based on available evidence and clinical experience, we favor CGM as a first-line technology recommendation for the treatment of type 1 diabetes (T1D).

**Electronic supplementary material:**

The online version of this article (10.1007/s11892-019-1177-7) contains supplementary material, which is available to authorized users.

## Introduction

The first commercially available insulin preparations varied in potency up to 25%, contained impurities associated with allergic reactions, and necessitated several injections daily due to non-physiologic pharmacokinetics [1]. The introduction of insulin analogues, with both rapid and prolonged durations of action, has been shown to reduce hypoglycemia and offer improved flexibility to patients [2]. These newer insulins have allowed for ways to more intensively administer insulin, either as multiple daily injections (MDI) or continuous subcutaneous insulin infusion (CSII) using an insulin pump, which have now been in clinical use for over 40 years. It is possible to achieve significant improvements in glycated hemoglobin (HbA1c) with either MDI or CSII. In the Diabetes Control and Complications trial (DCCT), the intensive group demonstrated major reductions in microvascular complication risk as compared with control [3]. In a subsequent analysis of the intensive group, nearly 40% were on CSII by the end of the DCCT. For those in the intensive group who only used CSII (*n* = 124) vs those who only used MDI (284), there was a slight favor towards CSII (final HbA1c of 6.8% with CSII vs 7.0% with MDI, *P* ≤ 0.05) [4]. A meta-analysis comparing MDI versus CSII regimens both utilizing rapid acting insulin analogues performed by Monami et al. demonstrated significant improvement of HbA1c in comparison with MDI (standardized difference in mean, − 0.3 [− 0.4; − 0.1]%; *P* < 0.001).The authors demonstrated no significant difference in the rate of severe hypoglycemic episodes [5]. Other meta-analyses have shown reduction in hypoglycemia, depending upon the definition used, and when including studies comparing CSII with MDI regimens utilizing regular human insulin [6, 7]. CSII also offers advantages over MDI in individuals with clinical considerations such as dawn phenomenon, extreme insulin sensitivity, erratic schedules, and needle phobias [8].

Historically physicians detected hyperglycemia by examining urine samples for volume, appearance, and taste. The first commercially available SMBG became available starting in the 1980s and CGM in 1999 [9]. A distinguishing characteristic of CGM is that it measures interstitial fluid (ISF) glucose concentration as opposed to SMBG, which measures capillary blood glucose concentration. Although ISF and plasma blood glucose concentration are highly correlated, there is a well-reported lag between ISF and plasma glucose concentration due to rate limited transport between vascular to intracellular compartments. Studies characterizing this temporal relationship report a delay between 4 and 50 min, with more recent evidence suggesting 7–8 min in persons with type 1 diabetes (T1D). Large, rapid fluctuations in plasma glucose concentration have been shown to accentuate this time lag [10, 11]. A meta-analysis published in 2013 demonstrated improved HbA1c with CGM use (mean change HbA1c − 0.2%), but mainly in patients starting sensor augmented pumps at the same time (mean change HbA1c − 0.7%). There was no significant difference as compared with SMBG in regard to episodes of severe hypoglycemia or ketoacidosis [12].

**Fig. 1:**
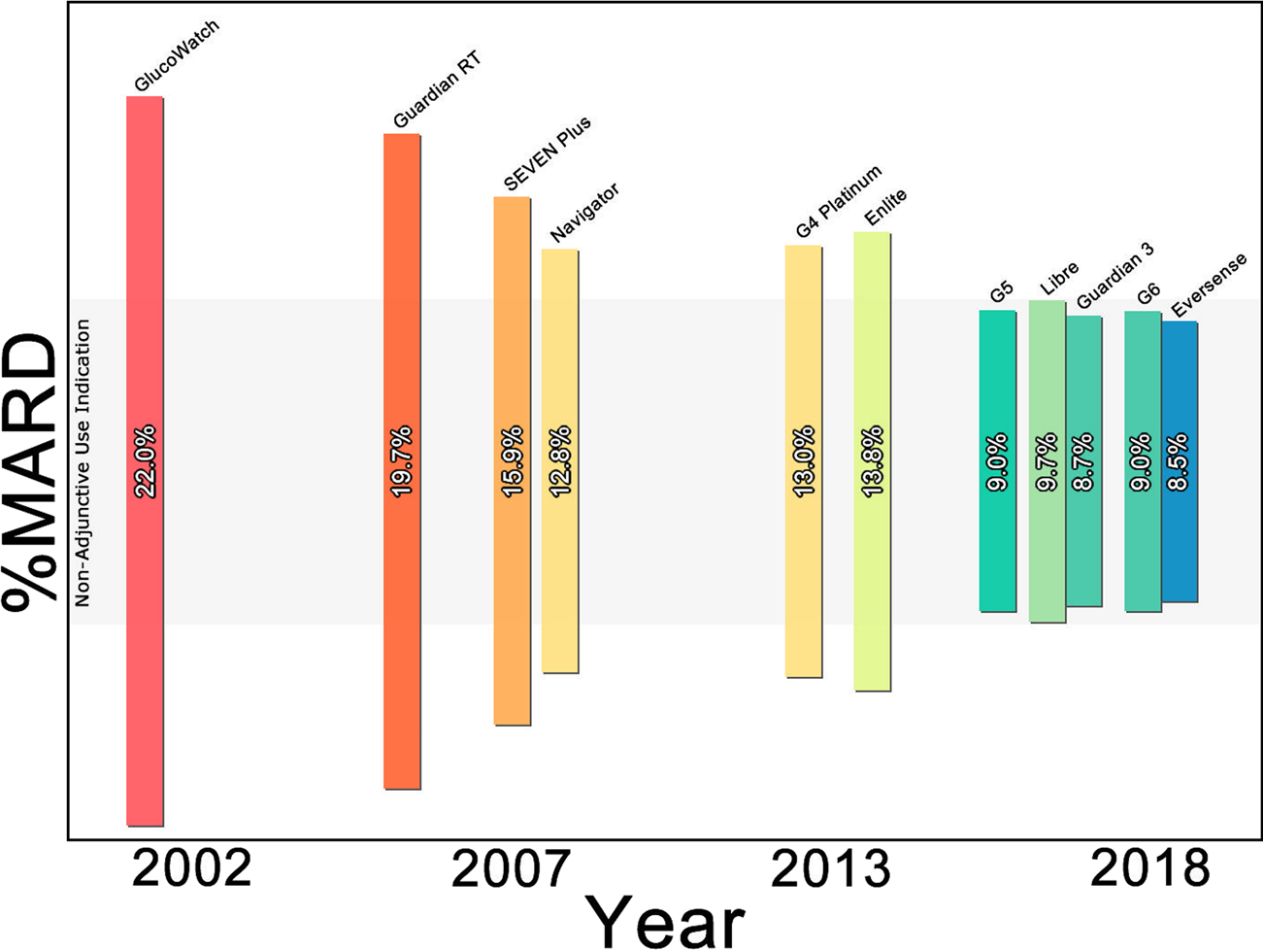
Device %MARD vs year commercially available

More accurate sensors combined with “smart” predictive algorithms have been shown to attenuate ISF glucose concentration dynamics [13]. Mean absolute relative difference (MARD) is frequently used to assess the accuracy of CGM devices. This measurement is the mean of the absolute difference expressed as a percentage of a reference glucose concentration over many samples [14]. Some of the first commercially available CGMs had a published MARD of > 20% [15]. The latest generation CGM devices have a MARD < 10% (Fig. 1, Supplemental Table 1). This is equivalent to or exceeds MARD of some SMBG devices and is felt safe by many sources for non-adjunctive use (i.e., treatment decisions) of CGM [16, 17]. Other significant advancements include extended sensor life and no required SMBG calibration measurements.

## Glucose Metrics

**Fig. 2:**
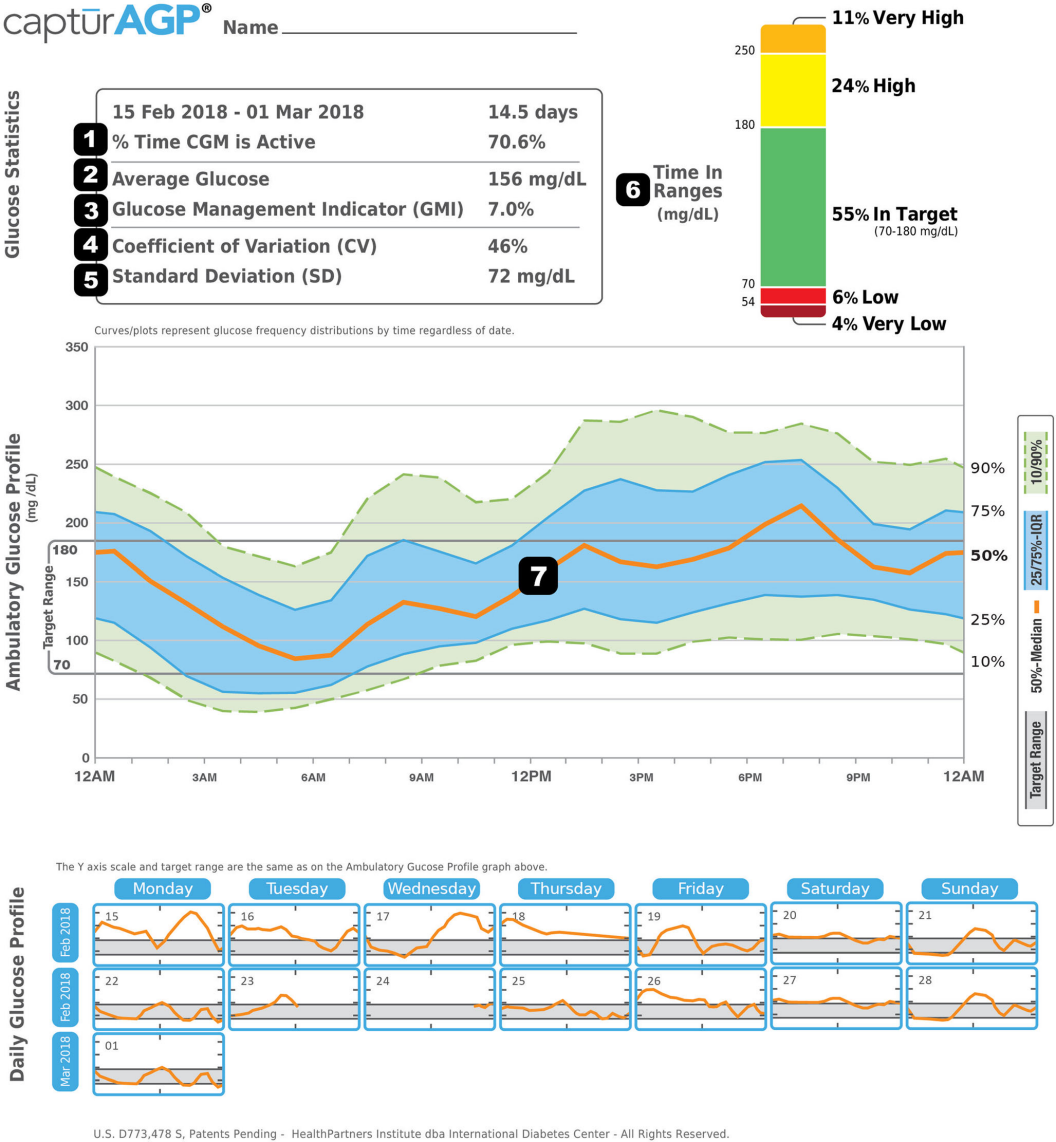
Representative Ambulatory Glucose Profile. (©2019 International Diabetes Center at Park Nicollet, Minneapolis, MN. Used with permission. See AGPreport.org for more information)

While HbA1c and SMBG may provide approximations of an individual’s glycemic management, glycemic variability and hypoglycemic burden can be less evident [18, 19]. The Advanced Technologies and Treatments for Diabetes (ATTD) Congress convened an international panel of expert physicians and researchers in February 2017 to define specific metrics for assessing CGM data. The consensus group recommended the use of a standardized single page glucose report of CGM data (Ambulatory Glucose Profile (AGP) developed at the International Diabetes Center in Minneapolis, MN) (Figure 2, Table 1) [20, 21].

**Table 1:** Definitions of CGM metrics present within the Ambulatory Glucose Profile

Key	Measure	Definition	Formula	Variables
1	Percent time CGM active	Hours the CGM collected data, divided by number of hours in the report	ti/*t*	ti = time cgm data collected, *t* = time in the report
2	Average glucose	All glucose values added together, divided by number of readings	∑xi/*k*	xi = individual glucose values, *k* = number of observations
3	Glucose Management Indicator	Calculated from average glucose; estimates future lab HbA1C	3.31 + 0.02392x̄	*x̄*=average glucose in mg/dL
4	Coefficient of variation	How far apart (wide) glucose values are; ideally a low number	*s*/*x̄*	*s* = standard deviation, *x̄*=average glucose
5	Standard deviation	How far values are from the average; ideally a low number	∑xi-*x̄*/2 *k*-1	xi = individual glucose values, *x̄*=average glucose, *k* = number of observations
6	Time in range	Hours the CGM measures glucose within a specified range, divided by number of hours in the report	tr/*t*	tr = time in range, *t* = time in the report
7	Ambulatory Glucose Profile	Daily glucose profiles are combined to make a 1 day (24-h) picture. Ideally, lines would stay within gray shaded area (target range)		Orange: median (middle) line where half of the glucose values are above and half are below; ideally, the orange line is mostly flat and inside the gray shaded area, Blue: area between blue lines shows 50% of the glucose values; ideally, space between is narrow, Green: 10% of values are above (90% top line) and 10% are below (10% bottom line); ideally, the closer the green lines are to the gray shaded area, the better

The Glucose Management Indicator (GMI) is calculated from average glucose and estimates future lab HbA1c. It is an approximation familiar to most clinicians and patients.

Time in range (TIR) represents the percentage of time measured within a specific glucose threshold. A value between 70 and 180 mg/dL is a generally accepted target as it has been shown achieving 70% of the values within this range achieves a HbA1c of approximately 7.0% [22]. TIR has additionally been correlated with reduction in microvascular complications [23]. Time spent with glucose < 70 mg/dL is a surrogate for hypoglycemic burden. This threshold is sufficiently low to recommend treatment with a fast-acting carbohydrate and or adjustment of glucose-lowering therapy [24].

Standard deviation (SD) and coefficient of variation (%CV) are measures of glycemic variability. Some controversy exists as to the importance of glycemic variability as a clinical target, but it has also been correlated with development of microvascular complications [25–27]. Glycemic variability has additionally been associated with increased risk of hypoglycemia, patient satisfaction, and quality of life measures [28–31].

## Recently Published Data Comparing SMBG with CGM

**Fig. 3:**
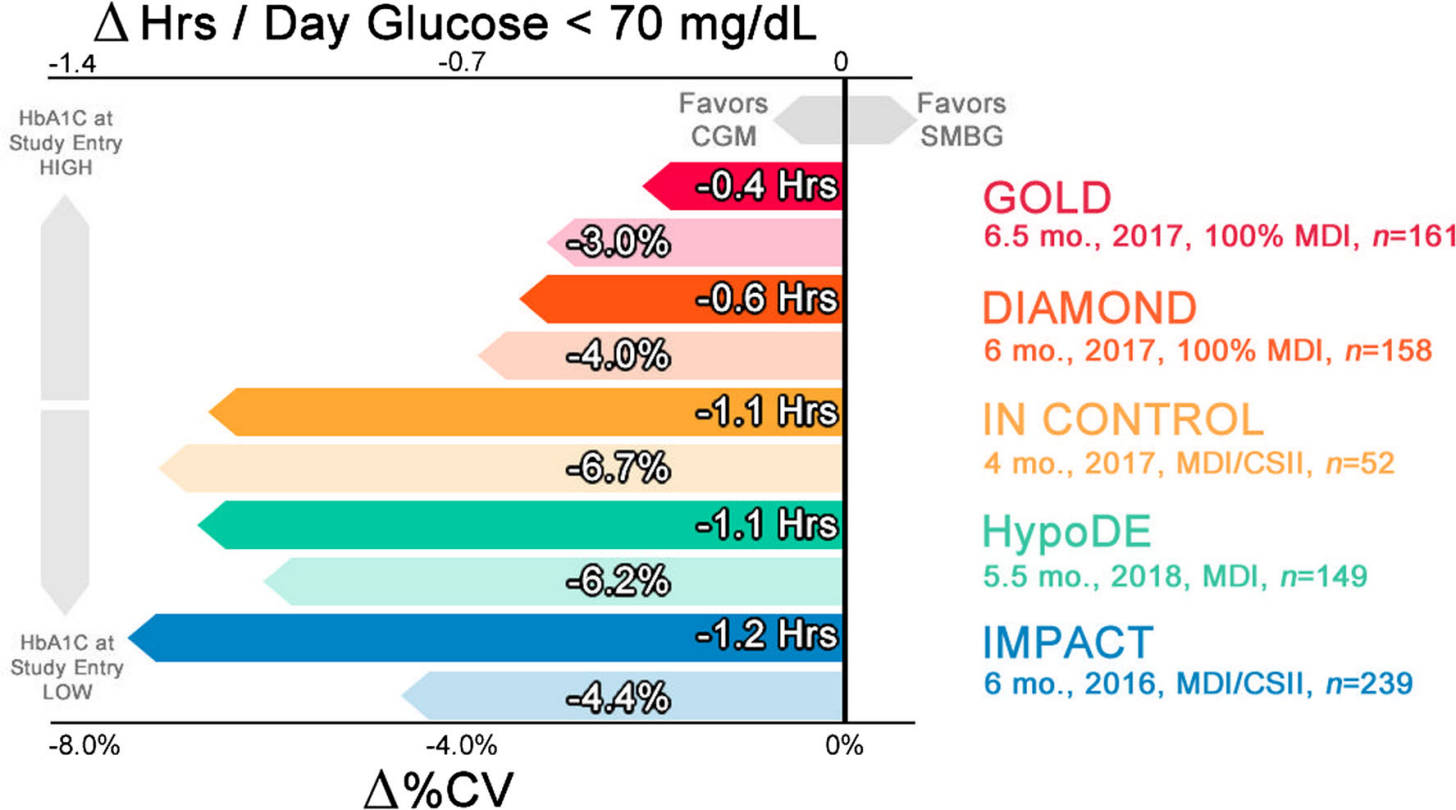
Summary of recently published CGM trials demonstrating reduction in time spent with glucose < 70 mg/dL and %CV as compared with SMBG

Recent studies of CGM vs SMBG associate CGM use with improved glycemic variability as measured by %CV and time spent with glucose < 70 mg/dL as compared with SMBG (Figure 3). Not all are associated with statistically significant improvement in HbA1c, which could be explained by low baseline HbA1c at study entry, reduction in hypoglycemia, and reductions in glucose variability not reflected in the HbA1c average.

The GOLD study enrolled 161 participants with T1D on MDI therapy and baseline HbA1c of 8.6% in a randomized cross-over study to either CGM or SMBG first. Treatment periods were 26 weeks each with 17-week washout periods between assignments. CGM use was associated with statistically significant improved HbA1c by − 0.4% (*P* < 0.001). CGM use was also associated with reductions in time spent with glucose < 70 mg/dL during the day by 40%, evening by 48% (*P* < 0.001), and %CV 37% vs 40% (difference − 3% (− 5 to − 2%) *P* < 0.001) [32, 33].

The DIAMOND study enrolled 158 participants with T1D on MDI therapy and baseline HbA1c of 8.6%, and randomized them to either CGM or SMBG. The treatment period was 24 weeks. CGM use was associated with statistically significant improved HbA1c by − 0.6% (*P* < 0.001). CGM use was also associated with statistically significant reductions in time spent with glucose < 70 mg/dL by 46% (*P* = 0.002) and %CV 38% vs 42% (difference − 4% (− 6 to − 2%) (*P* < 0.001) [34].

The IMPACT study enrolled 328 participants with T1D and baseline HbA1c 6.7% in a randomized trial of SMBG vs CGM. Seventy percent of participants utilized MDI and 30% CSII. The treatment period was 6 months. CGM use was not associated with statistically significant improved HbA1c. CGM use was associated with statistically significant reductions in time spent < 70 mg/dL by 38% (*P* < 0.0001) and %CV 37.6% vs 41.8% (difference − 4.4% (− 5.0 to − 3.8%) (*P* < 0.0001) [35].

## Recently Published Data Comparing SMBG with CGM in Individuals with Hypoglycemia Unawareness

The IN CONTROL study enrolled 52 participants with T1D and baseline HbA1c of 7.5% in a randomized cross-over trial to either SMBG or CGM first. Insulin delivery methods included 44% CSII and 56% MDI. Inclusion criteria were notable for hypoglycemia unawareness. Treatment periods were 16 weeks with a 12 week of washout period. No significant difference in HbA1c was observed. CGM use was associated with statistically significant reductions in time spent with glucose < 70 mg/dL by 40% (*P* < 0.001) and %CV 39.5% vs 46.3% (difference − 6.7% (− 8.0 to − 5.5%) *P* < 0.0001). It is also worth mentioning the authors did not demonstrate a difference for time spent in a normoglycemic state between MDI and CSII [36].

The HypoDE study enrolled 149 participants with T1D and baseline HbA1c 7.4% in a randomized trial of SMBG vs CGM. Inclusion criteria were notable for MDI therapy and hypoglycemia unawareness. The treatment period was 22 weeks. CGM was not associated with a statistically significant reduction in HbA1c. CGM was associated with statistically significant reductions in time spent with glucose < 70 mg/dL by 75% (*P* < 0.0001) and %CV 41.1% vs 34.1% (difference − 6.2% (− 7.5 to − 5.0%) *P* < 0.0001) [37].

## Recently Published Clinical Research Comparing CGM with CSII

There is some limited literature directly comparing CGM vs CSII.

A retrospective review of 396 newly diagnosed patients with T1D utilizing CSII and MDI was performed analyzing HbA1c, among other outcomes, in CGM users and non-CGM users over 2.5 years of follow-up. CGM users had a lower HbA1c regardless of insulin delivery method. Specifically, the MDI + CGM group had significantly lower HbA1c than the CSII only group (7.7% ± 0.2% vs 8.7% ± 0.07%, *P* < 0.0001) [38].

A similar cross-sectional analysis of 17,731 registry participants from the Type 1 Diabetes Exchange also showed improved HbA1c in CGM users regardless of insulin delivery method. Specifically, the MDI + CGM group had a significantly lower HbA1c than CSII alone users (7.7 ± 1.1% vs 8.3 ± 1.5%, adjusted *P* < 0.001) [39].

In a 28-week follow-on trial to the DIAMOND study, 75 participants utilizing CGM with baseline HbA1c 7.6% were randomly assigned to either continue MDI or transition to CSII. CSII users demonstrated increased time in range 70–180 mg/dL vs MDI (791 vs 741 min per day, *P* = 0.01). However, CSII users also demonstrated increased time spent with glucose < 70 mg/dL vs MDI (49 vs 32 min per week, *P* = 0.0001). The %CV was 39% in the CSII group and 37% in the MDI group, which did not achieve statistical significance (*P* = 0.25) [40•].

The COMISAIR study enrolled 65 participants with baseline HbA1c 8.3% randomized to either MDI + SMBG (*n* = 18), CSII + SMBG (*n* = 20), MDI + CGM (*n* = 12), or sensor-augmented pumps (SAP) (*n* = 15). Participants were followed for 1-year at 3-month intervals. The SAP and MDI + CGM groups demonstrated comparable outcomes with statistically significant reduction in both HbA1c (7.1% at end of study) and glycemic variability (total standard deviation at study entry 4.0 mmol/L vs 3.0 mmol/L at study completion; *P* < 0.0001). The CSII + SMBG group also demonstrated improved HbA1c 7.9% and glycemic variability (3.9 mmol/L vs 3.4 mmol/L), but the study authors remarked MDI + CGM was “clearly superior” to CSII + SMBG. No significant improvement was noted in any study endpoints by the MDI + SMBG group [41•].

## Practical Considerations

Starting CSII takes on average 2–4 h of initial patient education [42]. This includes device selection, configuration, canula insertion technique, and appropriate responses to device emergencies. Close follow-up is recommended during transition from MDI to CSII to confirm appropriate insulin dosing. A multi-disciplinary team consisting of an endocrinologist, diabetes educator, and dietician is also typically recommended. In contrast, starting CGM is often complementary to an existing treatment regimen. Patient education is more focused, emphasizing the difference between SMBG and CGM, device selection, configuration, and sensor insertion technique [43].

There is an association between CGM use and improved patient satisfaction and quality of life measures [32–37]. A recent survey of 1040 adolescents and their parents associated CGM use with reduced diabetes-specific emotional distress as compared with MDI alone, CGM + CSII, and CSII alone. The authors also reported CGM use was associated with lower HbA1C compared with MDI alone, CSII alone, and comparable with CGM + CSII. They hypothesized this was due to CGM making diabetes management easier with improved detection of hypoglycemia and increased time spent in range [44]. There is a well-established association between hypoglycemia and decreased quality of life scores [45]. Reducing hypoglycemic burden has clinical implications as antecedent hypoglycemia has been shown to impair future hypoglycemia awareness [46]. With improvement in hypoglycemia detection, CGM use has also been associated with recovery of hypoglycemia awareness. However, it should be noted CGM has not been found superior to SMBG in conjunction with structured patient education for recovery of hypoglycemia awareness [37, 47].

**Fig. 4:**
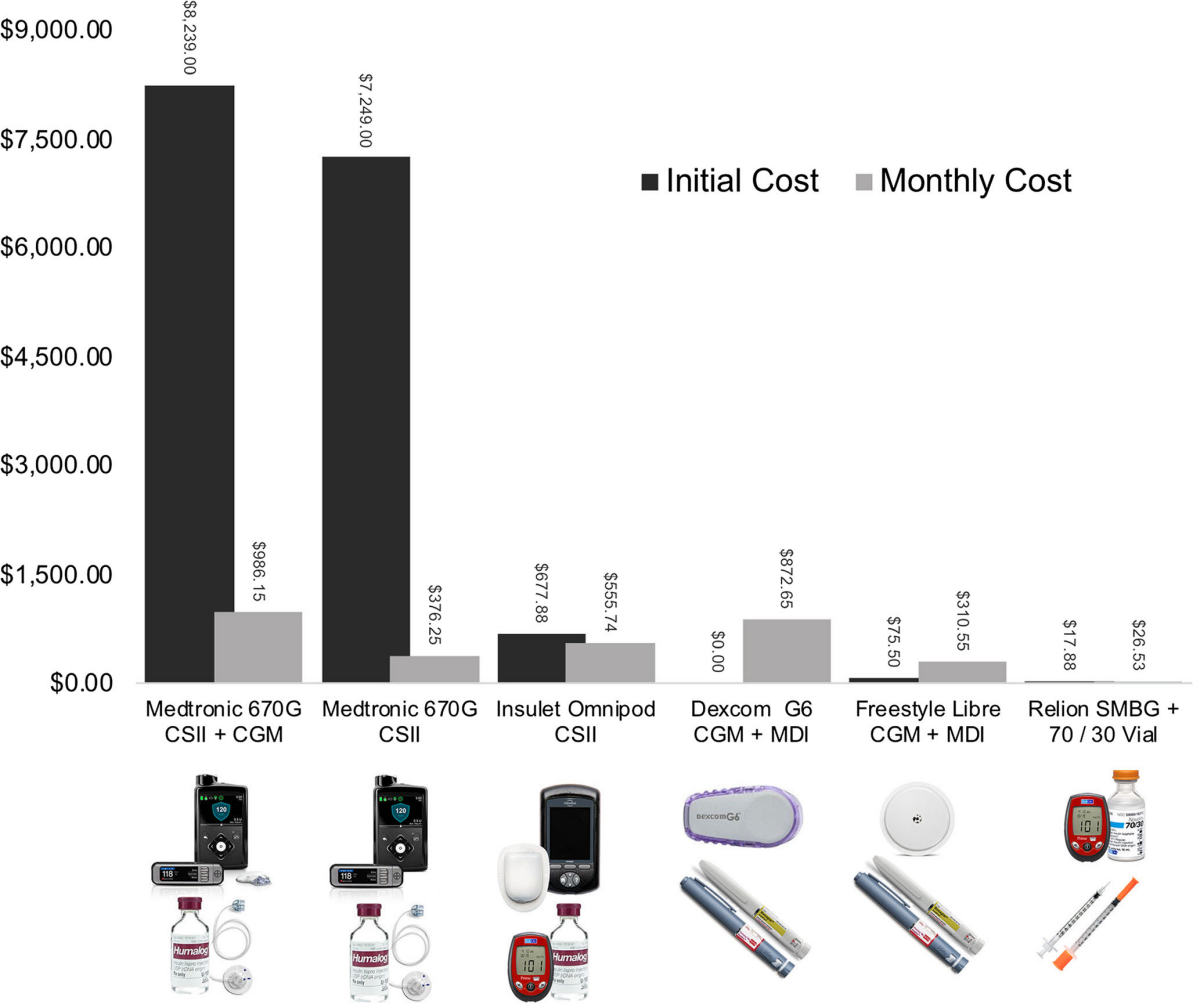
Estimated Initial and ongoing out-of-pocket costs of various diabetes treatment approaches. Refer to Supplemental Table 2 for pricing appendix

CGM has a lower up-front cost as compared with CSII (Figure 4, Supplemental Table 2). This is in large part due to the expense of the insulin pump itself. This cost is amplified by rapid device obsolescence as manufacturers frequently release new models with each incremental feature upgrade. Conversely, many CGMs integrate with patient owned smart devices circumventing the up-front cost of a dedicated receiver. Integration with smart devices enables functions previously only available on insulin pumps. These include hypoglycemia and hyperglycemia alarms, digital log books, carbohydrate counters, and insulin dosing calculators. Transfer of CGM data via the internet to cloud-based storage also facilitates telemedicine and family alerts.

## Conclusion

The centennial anniversary of the discovery of insulin will be reached in 2021 [1]. There have been many subsequent breakthroughs translated into routine clinical practice greatly improving the care of persons with T1D [48]. Advancements have been accelerated by validation that lower HbA1c with frequent SMBG is associated with delay or prevention of diabetes-related microvascular complications [3, 49]. The newest generation of CGMs offers improved accuracy and ease of use. They provide a more complete glycemic profile for patients and providers to make treatment decisions. Although earlier research focused on the addition of CGM to CSII, recent evidence demonstrates CGM use is associated with improved outcomes irrespective of insulin delivery method. Practical considerations such as patient satisfaction, initial education, and up-front costs of CGM are also frequently superior to CSII. For these reasons, we advocate for CGM as the initial technology recommendation. If additional technology is needed to improve time in target range or to further reduce time in hypoglycemic range, insulin pump therapy should be discussed with consideration for a hybrid closed loop system to achieve these goals [50–52].

## Electronic supplementary material

ESM 1 (DOCX 18 kb)

ESM 2 (DOCX 20 kb)

## Abbreviations

SMBG: Self-monitoring of blood glucose
CGM: Continuous glucose monitor
DM: Diabetes mellitus
T1D: Type 1 diabetes
T2D: Type 2 diabetes
%CV: Percent coefficient of variation
SD: Standard deviation
GMI: Glucose Management Indicator
TIR: Time in range
MDI: Multiple daily injections
CSII: Continuous subcutaneous insulin infusion
ISF: Interstitial fluid
HbA1c: Glycosylated hemoglobin
