# Supplementary material for: Advanced Technology in the Management of Diabetes: Which Comes First—Continuous Glucose Monitor or Insulin Pump?
Source: Curr Diab Rep. 2019 Jun 27;19(8):50. doi: 10.1007/s11892-019-1177-7 (PMC6597598; doi:10.1007/s11892-019-1177-7)
Supplement: Supplementary file 1 — (DOCX 18 kb) [file 11892_2019_1177_MOESM1_ESM.docx]

**Supplemental Table 1: Device %MARD Vs. Year Commercially Available Appendix Information**

| **Device** | **MARD** | **FDA Approval Date** | **FDA Approval Number** | **Source** |
| --- | --- | --- | --- | --- |
| GlucoWatch | 22% | 3/22/01 | P990026 | Diabetes Research in Children Network (DirecNet) Study Group. "The accuracy of the GlucoWatch® G2™ Biographer in children with type 1 diabetes: results of the Diabetes Research in Children Network (DirecNet) accuracy study." Diabetes technology & therapeutics 5.5 (2003): 791. |
| Guardian Real-Time | 19.70% | 7/18/05 | P980022 S011 | https://www.accessdata.fda.gov/cdrh_docs/pdf/P980022S011B.pdf |
| SEVEN plus | 15.90% | 5/31/07 | P050012 | Bailey, Timothy, Howard Zisser, and Anna Chang. "New features and performance of a next-generation SEVEN-day continuous glucose monitoring system with short lag time." *Diabetes technology & therapeutics* 11.12 (2009): 749-755. |
| Navigator | 12.80% | 3/12/08 | P050020 | Weinstein, Richard L., et al. "Accuracy of the 5-day FreeStyle Navigator Continuous Glucose Monitoring System: comparison with frequent laboratory reference measurements." *Diabetes care* 30.5 (2007): 1125-1130. |
| G4 Platinum | 13% | 10/5/12 | P120005 | Nakamura, Katherine, and Andrew Balo. "The accuracy and efficacy of the Dexcom G4 platinum continuous glucose monitoring system." *Journal of diabetes science and technology* 9.5 (2015): 1021-1026. |
| Enlite | 13.80% | 9/26/13 | P120010 | Keenan, Desmond Barry, et al. "Accuracy of the Enlite 6-day glucose sensor with guardian and Veo calibration algorithms." *Diabetes technology & therapeutics* 14.3 (2012): 225-231. |
| G5 | 9.00% | 8/19/15 | P120005 S033 | Bailey, Timothy S., Anna Chang, and Mark Christiansen. "Clinical accuracy of a continuous glucose monitoring system with an advanced algorithm." *Journal of diabetes science and technology* 9.2 (2014): 209-214. |
| Libre | 9.70% | 9/23/16 | P150021 | Klimek, Monika, and Tytus Tulwin. "Continuous glucose monitoring: review of promising technologies." *MATEC Web of Conferences*. Vol. 252. EDP Sciences, 2019. |
| Guardian 3 | 8.70% | 9/28/16 | P160017 | Josereyes. “Guardian™ Sensor 3.” Medtronic, 18 Dec. 2018, professional.medtronicdiabetes.com/guardian-sensor-3. |
| G6 | 9.00% | 3/27/18 | DEN170088 | Shah, Viral N., et al. "Performance of a factory-calibrated real-time continuous glucose monitoring system utilizing an automated sensor applicator." *Diabetes Technology & Therapeutics* 20.6 (2018): 428-433. |
| Eversense | 8.50% | 6/21/18 | P160048 | https://www.accessdata.fda.gov/cdrh_docs/pdf16/P160048B.pdf |
