# Supplementary material for: Advanced Technology in the Management of Diabetes: Which Comes First—Continuous Glucose Monitor or Insulin Pump?
Source: Curr Diab Rep. 2019 Jun 27;19(8):50. doi: 10.1007/s11892-019-1177-7 (PMC6597598; doi:10.1007/s11892-019-1177-7)
Supplement: Supplementary file 2 — (DOCX 20 kb) [file 11892_2019_1177_MOESM2_ESM.docx]

**Supplemental Table 2: Various Diabetes Treatment Approaches Pricing Appendix Information**

|  | **Medtronic 670G CSII + CGM** | **Medtronic 670G CSII** | **Insulet Omnipod CSII** | **Dexcom G6 CGM + MDI** | **Freestyle Libre CGM + MDI** | **Relion SMBG + 70 / 30 Vial** |
| --- | --- | --- | --- | --- | --- | --- |
| **Initial Cost** | $8,239.00 | $7,249.00 | $677.88 | $0.00 | $75.50 | $17.88 |
| Pump | $7,249.00 | $7,249.00 | - | - | - | - |
| Receiver / Glucometer / Transmitter | $990.00 | - | $677.88 | - | $75.50 | $17.88 |
| **Monthly Cost** | $986.15 | $376.25 | $555.74 | $872.65 | $310.55 | $26.53 |
| 30 days (10 cc) insulin | $177.87 | $177.87 | $177.87 | $195.68 | $195.68 | $26.53 |
| 30 days Glucose Testing Supplies | $660.39 | $50.49 | $17.88 | $490.00 | $114.87 | - |
| 30 Days Device Supplies | $147.89 | $147.89 | $359.99 | $186.97 | - | - |

| **Item** | **Qty** | **Cost** | **Source** | **date accessed** |
| --- | --- | --- | --- | --- |
| Medtronic 670g Insulin Pump with Contour Next Meter | 1 ea | $7,249.00 | [www.adwdiabetes.com](http://www.adwdiabetes.com/) | 1/23/19 |
| Medtronic Guardian III Transmitter | 1 ea | $990.00 | [www.adwdiabetes.com](http://www.adwdiabetes.com/) | 1/23/19 |
| Omnipod Pods | 30 days | $359.99 | [www.adwdiabetes.com](http://www.adwdiabetes.com/) | 1/23/19 |
| Omnipod Receiver | 1 ea | $660.00 | [www.adwdiabetes.com](http://www.adwdiabetes.com/) | 1/23/19 |
| Dexcom G6 Sensor | 30 days | $490.00 | [www.adwdiabetes.com](http://www.adwdiabetes.com/) | 1/23/19 |
| Dexcom G6 Transmitter | 90 days | $560.90 | [www.goodrx.com](http://www.goodrx.com/) | 1/23/19 |
| Freestyle Libre Receiver | 1 ea | $75.50 | www.goodrx.com | 1/23/19 |
| Freestyle Libre Sensor | 30 days | $114.87 | www.goodrx.com | 1/23/19 |
| Basaglar Kwikpen | 15 ml | $248.59 | [www.goodrx.com](http://www.goodrx.com/) | 1/23/19 |
| Humalog Kwikpen | 15 ml | $338.47 | [www.goodrx.com](http://www.goodrx.com/) | 1/23/19 |
| Humalog Vial | 10 ml | $177.87 | [www.goodrx.com](http://www.adwdiabetes.com/) | 1/23/19 |
| Novolin 70/30 | 10 ml | $26.53 | [www.goodrx.com](http://www.adwdiabetes.com/) | 1/23/19 |
| Contour Next Test Strips | 100 ea | $50.49 | [www.adwdiabetes.com](http://www.adwdiabetes.com/) | 1/23/19 |
| Relion Test Strips | 100 ea | $17.88 | [www.walmart.com](http://www.walmart.com/) | 1/23/19 |
| Relion Glucometer | 1 ea | $16.98 | [www.walmart.com](http://www.walmart.com/) | 1/23/19 |
| Medtronic Reservoir | 30 days | $34.99 | [www.adwdiabetes.com](http://www.adwdiabetes.com/) | 1/23/19 |
| Medtronic Quickset | 30 days | $112.90 | [www.adwdiabetes.com](http://www.adwdiabetes.com/) | 1/23/19 |
| Medtronic Guardian III Sensor | 30 days | $609.90 | [www.adwdiabetes.com](http://www.adwdiabetes.com/) | 1/23/19 |
